# Selegiline reduces daytime sleepiness in patients with Parkinson's disease

**DOI:** 10.1002/brb3.1880

**Published:** 2021-03-23

**Authors:** Marco Gallazzi, Marco Mauri, Maria Laura Bianchi, Giulio Riboldazzi, Lucia Princiotta Cariddi, Federico Carimati, Valentina Rebecchi, Maurizio Versino

**Affiliations:** ^1^ Neurologia e Stroke Unit ASST Sette Laghi Ospedale di Circolo di Varese Italy; ^2^ Università dell’Insubria Varese Italy; ^3^ Casa di Cura Fondazione Gaetano e Piera Borghi Brebbia Italy; ^4^ Neurologia e Stroke Unit ASST Valle Olona Ospedale S. Antonio Abate Gallarate Italy

**Keywords:** Parkinson’s disease, Selegiline, sleepiness

## Abstract

**Objectives:**

Excessive daytime sleepiness (EDS) affects a large percentage of Parkinson's disease (PD) patients, and it is enhanced by dopamine agonist drugs. Currently, there is no treatment of choice for EDS in PD. Our aim was to check the clinical impression that some patients who were given selegiline, a selective inhibitor of monoamine oxidase B, experienced an improvement in their daytime somnolence.

**Methods:**

In the present study, we retrospectively identified 45 Parkinson's disease patients (21 females and 24 males) among those referred to the PD Center in Varese that (a) showed excessive daytime sleepiness, usually developed after the introduction of a dopamine agonist, (b) were given selegiline 10 mg to improve their treatment schedule independently of excessive sleepiness, and (c) in whom the Epworth Sleepiness Scale (ESS) and the Parkinson's Disease Sleep Scale (PDSS) scores were available both before and 3 months after the introduction of selegiline.

**Results:**

We compared the corresponding scores (ESS, PDSS, and UPDRS III) evaluated before and 3 months after the introduction of selegiline by the nonparametric Mann–Whitney U test: The differences showed a statistically significant improvement of somnolence but no change in the UPDRS III scores.

**Conclusion:**

Despite some limitations, our data suggest that selegiline may be a valuable add‐on therapy in PD patients to reduce their daytime somnolence.

## Graphical abstract

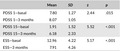

## 1 INTRODUCTION

Sleep disturbances are one of the most common and disabling nonmotor manifestations of Parkinson's disease (PD), affecting almost 80% of patients (Shen et al., 2018). Disrupted sleep–wake cycles contribute to poor quality of life and increased risk for accidents, leading to increased morbidity and mortality in the PD population (Rocha et al., 2015). The exact pathophysiology of sleep–wake disturbances in PD remains largely unknown, but the etiology is likely to be multifactorial, including the impact of motor symptoms on sleep, primary sleep disorders (sleep apnea and REM behavior disorder), adverse effects of medications, and neurodegeneration of central sleep–wake regulatory systems (Rocha et al., 2015). As a consequence of poor sleep, PD patients complain of daytime disturbances (excessive daytime sleepiness and sleep attacks) also.

Daytime sleepiness is defined as excessive daytime sleepiness (EDS) when it causes a subjective complaint or interferes with function. The International Classification of Sleep Disorders, third edition (ICSD‐3), defines EDS as the inability to maintain wakefulness and alertness during the major waking periods of the day, with sleep occurring unintentionally or at inappropriate times almost daily for at least three months (Sateia, 2014).

Mechanisms underlying EDS are probably multiple: the lack of dopamine, the use of dopamine receptor agonists, the lack of serotonin and norepinephrine, autonomic dysfunction, and the loss of circadian rhythm and the hypocretin pathway (Videnovic et al., 2014).

There are several methods to evaluate EDS such as the Epworth Sleepiness Scale (ESS), the Parkinson's Disease Sleep Scale (PDSS), the multiple sleep latency test, and polysomnography.

EDS affects a large percentage of PD patients, and it is considered to be a possible preclinical marker of the disease itself (Abbott et al., 2005), although EDS is related to disease duration (Shen et al., 2018), and the occurrence of EDS in idiopathic REM behavior disorder does not predict the conversion to PD (Postuma et al., 2017). The therapies used to treat the motor symptoms contribute to the onset of EDS, but Tholfsen et al. (2015) have recently documented a higher frequency of EDS in PD patients not yet treated compared to controls, and the occurrence of EDS increased with the progression of the disease. In this study, the main risk factor was an early predisposition to sleepiness as measured by ESS, and there was an association between EDS and male gender, the use of dopamine agonists, a high UPDRS‐ADL value, and depression Tholfsen et al. (2015).

Given the strong impact of EDS, many randomized controlled trials tested different types of possible treatments in these patients: modafinil (Hogl et al., 2002; Ondo et al., 2005; Rodrigues et al., 2016), caffeine (Noyce et al., 2012; Postuma et al., 2012), methylphenidate (Devos et al., 2007), istradefylline (Du & Chen, 2017; Suzuki et al., 2017), and memantine (Ondo et al., 2011), but none of them proved their effectiveness (Seppi et al., 2011; Shen et al., 2018); sodium oxybate showed promising results, but it cannot be considered a first‐choice drug because it is a depressant of the nervous system and respiratory system and has a potential for addiction (Ondo et al., 2008).

Selegiline is a selective inhibitor of monoamine oxidase B (MAO‐B): Monoamine oxidases (MAOs) are intracellular enzymes located on the mitochondrial membrane, and MAO‐B is the main enzyme responsible for the catabolization of dopamine. It has been documented (Robottom, 2011) that selegiline can be safely used in PD patients, because it has a symptomatic effect when used as monotherapy, can delay the introduction of levodopa, in patients with motor fluctuations is able to reduce the off‐time and improve the wearing‐off, and it may have a neuroprotective effect (Takahata et al., 2006). Selegiline can be regarded as an old drug, but its efficacy is comparable to that of more recent MAO‐B inhibitor, rasagiline (Cereda et al., 2017; Marconi & Zwingers, 2014).

Selegiline is metabolized to demethyl‐selegiline and L‐methamphetamine, which in turn can be further metabolized to L‐amphetamine and other minor metabolites, but there is no evidence about the possibility of selegiline‐induced dependence (Schneider et al., 1994). However, one of the reasons for the use of rasagiline, another MAO‐B inhibitor, is that it does not have amphetamine‐like metabolites (Knudsen Gerber, 2011). Finally, selegiline can be considered, not as a first‐line drug, for the treatment of hypersomnias of central origin (Morgenthaler et al., 2007).

In our experience, some patients who were given selegiline, mostly because rasagiline proved to be not so effective, reported that selegiline improved their daytime somnolence.

In this scenario, we retrospectively tested in a group of 45 PD patients the effect of selegiline on excessive daytime sleepiness.

## 2 MATERIALS AND METHODS

We selected a population of 45 (21 females and 24 males) Parkinson's disease patients among those referred to the PD Center in Varese. The diagnosis of PD was made according to UK Parkinson's Disease Society Brain Bank clinical diagnostic criteria (Hughes et al., 1992). The clinical features of the patients are shown in Table 1.

**Table 1:** Clinical features. UPDRS III: Unified Parkinson's Disease Rating Scale III score

	Min	Max	Mean	*SD*
Age (yrs)	48	79	65.44	7.372
Disease duration (yrs)	3	11	6.76	2.258
L‐DOPA (mg)	0	850	461.11	268.389
L‐DOPA equivalent (mg)	210	1,170	719.58	264.46
UPDRS III	8	28	16.00	6.183
H&Y	1	2	1.84	0.367
MMSE	25	30	28.36	1.190

In our patients, the occurrence of sleep disorders, including day time sleepiness, is routinely checked for and acknowledged by using the Italian version of both the Epworth Sleepiness Scale (ESS) (Vignatelli et al., 2003) and the Parkinson's Disease Sleeping Scale (PDSS) (Pellecchia et al., 2012). The ESS lists 8 conditions for which the patient must rate the likelihood to fall asleep between 0 (never) and 4 (very likely); a total score greater than 10 suggests an excessive daytime sleepiness. The PDSS is a 15‐item visuo‐analog scale to investigate the sleep habit and quality in PD patients: Here, we considered item #1 (The overall quality of your night's sleep is?) and item #15 (Have you unexpectedly fallen asleep during the day?). We retrospectively identified the patients that 1) showed excessive daytime sleepiness, usually developed after the introduction of the dopamine agonist; excessive daytime sleepiness was defined to be so on the basis of the subjective complaint and an ESS score greater than 10 and/or a PDSS #15 less than 6. 2) Patients were given selegiline (10 mg) to improve their treatment schedule, usually as a substitute of rasagiline, but not because they complained of excessive sleepiness, and the patients were unaware about the possible effect of selegiline on their sleepiness 3) in whom the ESS and PDSS scores were available both immediately before and 3 months after the introduction of selegiline.

### 2.1 Statistical analyses

We compared the corresponding scores (ESS, PDSS #1, PDSS #15, and UPDRS III) evaluated before and 3 months after the introduction of selegiline by the nonparametric Mann–Whitney U test.

We also checked a significant possible correlation between the 3 months and basal difference of the ESS. PDSS #1 and PDSS #15 with the and disease duration, L‐DOPA equivalent dosage, basal UPDRS, and MMSE by the nonparametric Pearson correlation coefficient.

The significant level for all analyses was set at *p* = .01.

### 2.2 Statement of ethics

The data of this study were collected retrospectively, but all procedures performed were in accordance both with clinical and with the ethical and privacy standards of the institutional ethical committee and with the 1964 Helsinki Declaration. It was not possible to collect an informed consent to use their data from the participants.

## 3 RESULTS

We compared the scores from the three scales before and after 3 months of selegiline treatment and the differences were invariably significantly different (Table 2), with a borderline significance for the PDSS score. The self‐perceived quality of sleep (PDSS #1) improved, while both the tendency to fall asleep (PDSS #15) and the sleepiness during the daytime (ESS) decreased.

**Table 2:** Mann–Whitney U tests for the comparison of the Parkinson's Disease Sleeping Scale (PDSS) and of Epworth Sleepiness Scale (ESS) at different time points (basal recording and after 3 months)

	Mean	*SD*	z	*p*
PDSS 1—basal	7.80	1.27	2.44	.015
PDSS 1—3 months	8.07	1.05		
PDSS 15—basal	1.91	1.52	5.52	<.001
PDSS 15—3 months	6.18	2.33		
ESS—basal	12.96	4.22	5.17	<.001
ESS—3 months	7.91	4.26		

In the same time period, the UPDRS III score did not change significantly, since the mean value moved from 6.2 to 6.1.

Concerning ESS, a score more than 10 suggests the occurrence of a clinically relevant somnolence. At basal evaluation, 30 of 45 patients showed a score larger than 10; after the introduction of selegiline, the score improved in 41 patients, kept stable in 2, and worsened in 2 patients, and it was still greater than 10 in only 10 patients. The PDSS #15 score improved in all the patients.

We considered the possible correlation between the basal values of the three scores and the differences between the basal and the three months values of these scores and the other clinical variables, namely the age, the disease duration, the UPDRS III and the H‐Y scores, the L‐DOPA dosage, and the dopaminergic drugs L‐DOPA equivalent values, and no correlations proved to be significant.

## 4 DISCUSSION

We used the ESS and the PDSS scores to evaluate whether the introduction of selegiline could affect the sleep of 45 patients suffering from IPD.

Before the introduction of selegiline, the patients did not complain of their sleep during the night, but of involuntary falling asleep or of excessive sleepiness during the day; about this latter, 30 of our 45 patients showed an ESS score higher than 10. None of these scores were correlated with the clinical variables, namely with the duration or the severity of the disease or the dosage of L‐DOPA or of the dopaminergic drugs.

After the introduction of selegiline, the median values of all the scores improved, in particular item #15 of PDSS and the ESS score; about ESS, the score improved in 41 patients, kept stable in 2, and worsened in 2 patients, and it was greater than 10 in only 10 patients; again the degree of this improvement was not correlated with clinical variable. It is noteworthy that many of our patients switched from rasagiline to selegiline, and it is likely that the reduction in daytime somnolence occurred not because of the antiparkinsonian effect of these drugs rather than that of the L‐amphetamine metabolite of selegiline. Our study has some limitations since it is a retrospective, not controlled study, and selegiline was not tested against any other drug or placebo. Moreover, none of our patients had a significant cognitive decline (Table 1) and we are not confident that our findings can be safely applied in patients with a cognitive impairment. However, the patients were included because they presented, but not spontaneously complained of, daytime somnolence, and they did not expect that the introduction of selegiline in their treatment schedule was aimed to any particular benefit on their sleep/somnolence. In conclusion, our data suggest that selegiline may be a valuable add‐on therapy in PD patients without cognitive decline to reduce their daytime somnolence.

## CONFLICT OF INTEREST

Maurizio Versino received a financial support for research activities by Chiesi Pharmaceutical.

## AUTHOR CONTRIBUTION

GR and MLB conceived the study, and drafted and revised the manuscript. MG collected the data, and drafted and revised the manuscript. MM, LPC, FC, and VR drafted and revised the manuscript. MV analyzed the data, and drafted and revised the manuscript.

### PEER REVIEW

The peer review history for this article is available at https://publons.com/publon/10.1002/brb3.1880.

## Data Availability

The data that support the findings of this study are available from the corresponding author upon reasonable request.
